# Integrating multi-omics and machine learning to explore the role of amino acid metabolism in intervertebral disk degeneration

**DOI:** 10.3389/fneur.2026.1808282

**Published:** 2026-05-13

**Authors:** Xusheng Li, Ahmad Nazrun Shuid, Mohd Fairudz Mohd Miswan, Xiao Zhang, Wenbo Gu, Donghui Cao, Jungang Wang, Ziyang Jiang, Haifeng Yuan

**Affiliations:** 1 Department of Spinal Orthopaedics, General Hospital of Ningxia Medical University, Yinchuan, China; 2 Faculty of Medicine, Universiti Teknologi MARA, Sungai Buloh Campus, Jalan Hospital, Sungai Buloh, Selangor, Malaysia

**Keywords:** amino acid metabolism, biomarkers, *in silico* knockout, intervertebral disk degeneration, machine learning

## Abstract

**Objective:**

Intervertebral disk degeneration (IDD) is the leading cause of chronic low back pain, yet its link to amino acid metabolic reprogramming remains unclear.

**Methods:**

Three GEO transcriptomes were integrated; amino-acid-metabolism genes were intersected with differentially expressed genes. Core genes were selected by LASSO, SVM and random forest, incorporated into an SHAP-interpretable nomogram, and tested by single-cell analysis, *in silico* knockout, docking and WB.

**Results:**

Forty-three altered amino acid metabolism-related genes were identified, from which five core genes were screened: CETP, AIFM1, and GM2A were up-regulated; PNPLA2 and AGK were down-regulated. The constructed nomogram prediction model achieved an AUC value of 0.812. Degenerated intervertebral disks exhibited increased immune infiltration; the core genes either suppressed protective matrix genes or impaired stress defense capability. Molecular docking results showed that NVP-AEW541 and EGCG could bind to the AIFM1 protein with a binding free energy of −10.7 kcal/mol; WB confirmed protein trends.

**Conclusion:**

The five-core-gene signature is strongly associated with IDD and may represent a key regulatory pathway, offering a promising diagnostic model and potential therapeutic targets.

## 1 Introduction

Intervertebral disk degeneration (IDD) is the foremost pathological basis of chronic low back pain. With the intensifying global trend of population aging, it has become an increasingly severe public health challenge, imposing a heavy socioeconomic burden (1, 2). The occurrence of IDD is a complex biological process involving various cell types and molecular mechanisms, including apoptosis, inflammatory responses, and extracellular matrix (ECM) degradation (3). Currently, clinical management of IDD faces bottlenecks. When conservative treatments fail or disk degeneration causes severe neurological compression symptoms, surgical intervention becomes necessary (4). This mainstream treatment primarily focuses on symptom relief and fails to effectively halt or reverse the cascade of pathological degeneration (5).

In recent years, research has gradually revealed that cellular metabolic dysregulation plays a central driving role in the pathogenesis and progression of IDD (6). As fundamental units of cellular function, amino acids are not only building blocks for protein synthesis but also crucial regulators of energy metabolism, redox balance, and signal transduction (7, 8). Accumulating evidence indicates that imbalances in amino acid metabolism are closely linked to various chronic degenerative diseases such as osteoarthritis and neurodegenerative disorders (9–11). In the intervertebral disk microenvironment, nucleus pulposus cells endure prolonged physiological stress from hypoxia and low nutrient supply. Their metabolic patterns, especially the amino acid and lipid metabolic networks, are vital for maintaining cell viability, synthesizing extracellular matrix (ECM), and resisting apoptosis (12, 13). However, it remains unclear whether specific amino acid metabolic reprogramming occurs during IDD progression and how key genes may influence cell fate and the microenvironment.

**Figure 1:**
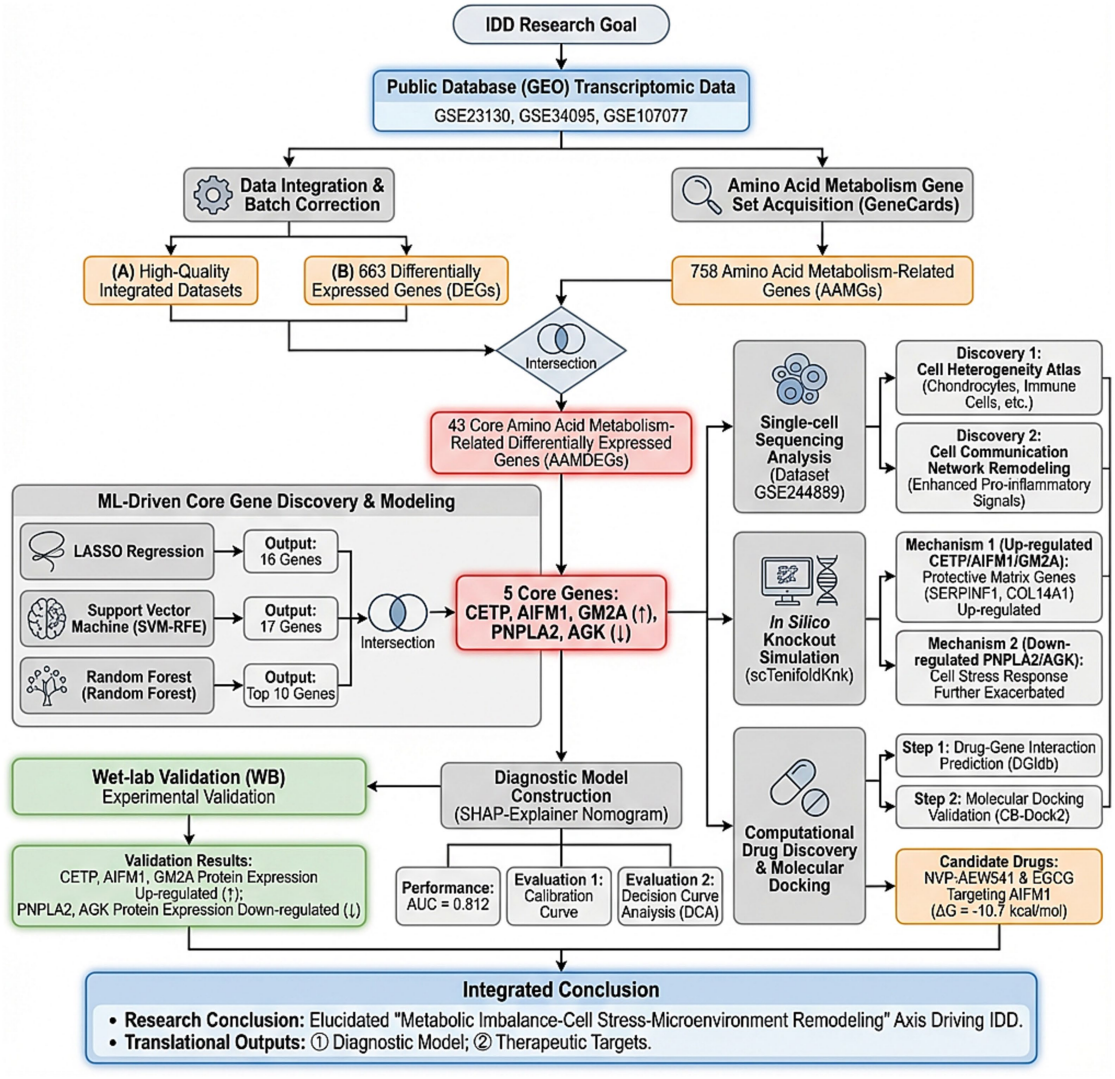
Flow chart of this study.

The central hypothesis of this study is that nucleus pulposus cells in IDD undergo characteristic reprogramming of amino acid metabolism. These changes can be encoded by a set of core metabolic genes and serve as biomarkers for early diagnosis and potential therapeutic targets. To this end, we integrated multi-omics strategies with computational biology methods. First, we systematically screened dysregulated amino acid metabolism-related genes in IDD through integrated analysis of public transcriptomic datasets (14). Subsequently, we employed multiple machine learning algorithms (LASSO regression, random forest, and support vector machine) for cross-validation to identify the most diagnostically valuable core gene set and utilized SHAP (Shapley Additive Explanations) methods for model interpretability analysis to quantify each gene’s contribution (15). To deeply dissect the biological functions of these core genes, we introduced single-cell transcriptomic analysis to clarify their cell type-specific expression patterns and explored their roles in constructing the degenerative microenvironment through cell communication analysis. More importantly, we used *in silico* gene knockout technology to simulate the impact of key gene functional loss on the global regulatory network, thereby systematically assessing their potential “driving” or “protective” roles (16). Finally, we explored potential therapeutic strategies targeting these key metabolic nodes through virtual screening and molecular docking of drug-gene interactions (17). The workflow of this study is illustrated in Figure 1.

## 2 Materials and methods

### 2.1 Data sources

Transcriptomic datasets of intervertebral disks from patients with IDD were retrieved from the GEO database, including GSE23130, GSE34095, and GSE107077 (18, 19). In the GSE23130 dataset, the experimental group comprised 8 IDD patients and the control group 15 subjects. The GSE34095 dataset included 3 IDD patients and 3 controls. The GSE107077 dataset consisted of 9 patients and 10 controls. In total, we obtained 20 experimental and 28 control samples. Tissue classification followed the Pfirrmann grading system (20): grades I-III were classified as mild degeneration (control group), and grades IV-V as severe degeneration (experimental group). Datasets were subsequently integrated, and batch effects were removed. All processing was conducted in the R 4.3.1 environment. Amino acid-related genes were obtained from the GeneCards database^1^ using “amino acid metabolism” as the query term, with “protein-coding” and “relevance score >30” as filtering criteria (21).

### 2.2 Differential expression gene analysis

The R package “limma” was used to detect differentially expressed genes (DEGs), with a threshold of *p* < 0.05 for screening DEGs between the IDD experimental and control groups (22). All processing was conducted in the R 4.3.1 environment. The key R packages and their versions used were: ‘limma’ (3.56.2), ‘pheatmap’ (1.0.12), ‘ggplot2’ (3.4.3), ‘clusterProfiler’ (4.8.3), ‘org. Hs.eg.db’ (3.17.0), ‘glmnet’ (4.1–8), ‘e1071’ (1.7–14), ‘randomForest’ (4.7–1.1), ‘rms’ (6.7–1), and ‘Seurat’ (4.3.0) (23–25). Finally, Gene Ontology (GO), Kyoto Encyclopedia of Genes and Genomes (KEGG), and Gene Set Variation Analysis (GSVA) were performed for gene and genome enrichment analysis, with *p* < 0.05 considered statistically significant.

### 2.3 Identification of core genes and related analyses

The R packages “glmnet,” “e1071,” and “randomForest” were used to perform LASSO regression, SVM, and random forest analyses on the DEGs in the disease group, respectively (26–28). These three machine learning algorithms were employed as feature selection tools to robustly and objectively identify the most informative and generalizable key genes from the amino acid metabolism-related differentially expressed genes, which exhibited potential collinearity. For the random forest, genes with top 10 importance scores were selected. The genes identified by all three algorithms were intersected, and the overlapping genes were defined as core genes and visualized in a Venn diagram. This feature selection step was designed for dimensionality reduction and noise elimination, rather than for constructing the final clinical prediction model. Boxplots were generated based on their expression levels. Furthermore, to determine the chromosomal location of the core genes, the NCBI database was queried.

### 2.4 SHAP-based interpretability analysis

To assess the contribution of each gene feature to the prediction outcome, this study employed the SHAP (Shapley Additive Explanations) method for model interpretation analysis (29). First, the dataset was randomly split into training and test sets at a 7:3 ratio, and multiple machine learning models were constructed for training and validation. The SHAP analysis was performed on an explanatory machine learning model—specifically, a random forest model—which we trained prior to constructing the final nomogram. The random forest model was selected as the “surrogate model” for SHAP calculation because it demonstrated the best discriminative ability on the test set. Subsequently, the permshap function was used to calculate the SHAP value for each gene on the test set, quantifying its marginal contribution to the model output. The purpose of this SHAP analysis was to conduct an in-depth, interpretable exploration of gene importance, capturing non-linear contributions and interactions among the five core genes. This approach is complementary to, rather than a substitute for, the final logistic regression-based nomogram model constructed for clinical application. Results were visualized using the shapviz package (30).

### 2.5 Prediction model construction

This study constructed and evaluated a nomogram model based on gene expression data for disease risk prediction (31). The nomogram was generated using the nomogram function. To assess the model’s calibration performance, a calibration curve was plotted using the calibrate function. Additionally, decision curve analysis (DCA) was performed using the decision_curve function.

### 2.6 Single-cell RNA sequencing data processing and analysis

The publicly available single-cell transcriptomic dataset GSE244889 was used, which includes nucleus pulposus tissue samples from 3 patients with severe disk degeneration and 4 with mild degeneration. Raw gene expression matrices were downloaded from the GEO database. Quality control and preprocessing were performed using R software and the Seurat package (v4.0). Quality control criteria were set as: at least 500 expressed genes detected per cell and mitochondrial gene percentage below 10%. Expression data were normalized using the “LogNormalize” method and further subjected to nonlinear dimensionality reduction and visualization via t-SNE. Cell clusters were annotated using known marker genes for nucleus pulposus tissue cell types. Cell–cell communication in IDD was analyzed using CellChat (version 1.6.1).

### 2.7 *In silico* gene knockout analysis

To investigate the mechanistic role of differentially expressed molecules identified by prior Western Blot analysis in intervertebral disk degeneration, this study conducted computational prediction method using the scTenifoldKnk algorithm. For the three molecules upregulated in degenerated tissues (CETP, AIFM1, and GM2A), their normal function was simulated to be knocked out to explore whether their overexpression participates in and drives the degenerative process. For the two key downregulated molecules in degenerated tissues (PNPLA2, AGK), further functional loss was simulated to assess their potential aggravating effect on disease progression. The analysis utilized quality-controlled single-cell transcriptomic data to construct a gene regulatory network. By simulating the knockout of the aforementioned target genes, the impact on the entire transcriptional network was systematically evaluated, thereby computationally verifying the biological significance of these molecules as key regulatory nodes in disk degeneration.

### 2.8 Drug prediction and molecular docking

To explore the potential of core metabolic genes as drug targets, this study conducted systematic computational drug discovery and virtual screening. First, the five core genes (CETP, AIFM1, GM2A, PNPLA2, and AGK) were submitted to the Drug-Gene Interaction Database (DGIdb) to predict molecules with known or potential interactions. For each core gene, we retrieved all candidate drugs with reported interaction evidence and ranked them based on the number of supporting evidences and significance level (*p*-value) provided by the database. The top-ranked candidate drugs for each gene were selected for subsequent molecular docking validation. For the selected candidate drugs and their corresponding predicted target proteins, semi-flexible molecular docking was performed using the CB-Dock2 online platform^2^. Blind docking mode was employed to assess binding feasibility. The strength of the binding interaction was represented by the binding free energy (ΔG, kcal/mol). Lower (more negative) binding energy indicates greater stability of the drug-target complex and stronger interaction.

### 2.9 Western blot (WB)

This study was approved by the Ethics Committee, and all sample collections adhered to the principles of the Declaration of Helsinki (32). Based on preoperative lumbar MRI Pfirrmann grading, 5 patients with grades I-II were included in the control group and 5 patients with grades III–V in the experimental group. Nucleus pulposus tissues obtained intraoperatively were homogenized in RIPA lysis buffer containing protease inhibitor to extract total protein, which was quantified using the BCA assay. Thirty micrograms of protein per lane was loaded onto 10% SDS-PAGE gels and transferred onto PVDF membranes via wet transfer. After blocking with 5% non-fat milk for 2 h, membranes were incubated overnight at 4 °C with the following primary antibodies: CETP (1:1000, Affinity, DF13606), PNPLA2 (1:1000, Affinity, DF8186), AGK (1:1000, Proteintech, 16,207-1-AP), AIFM1 (1:1000, CST, 5318S), GM2A (1:1000, Proteintech, 16,840-1-AP), and β-Actin (1:5000, Proteintech, 66,009-1-Ig). After washing with TBST, membranes were incubated with corresponding HRP-conjugated secondary antibodies (1:5000, Affinity) for 2 h, followed by ECL detection and imaging using a Tanon 5,200 system. Band intensities were analyzed with ImageJ and normalized to β-actin. Experiments were independently repeated three times.

## 3 Results

### 3.1 Acquisition and screening of differentially expressed genes

**Figure 2:**
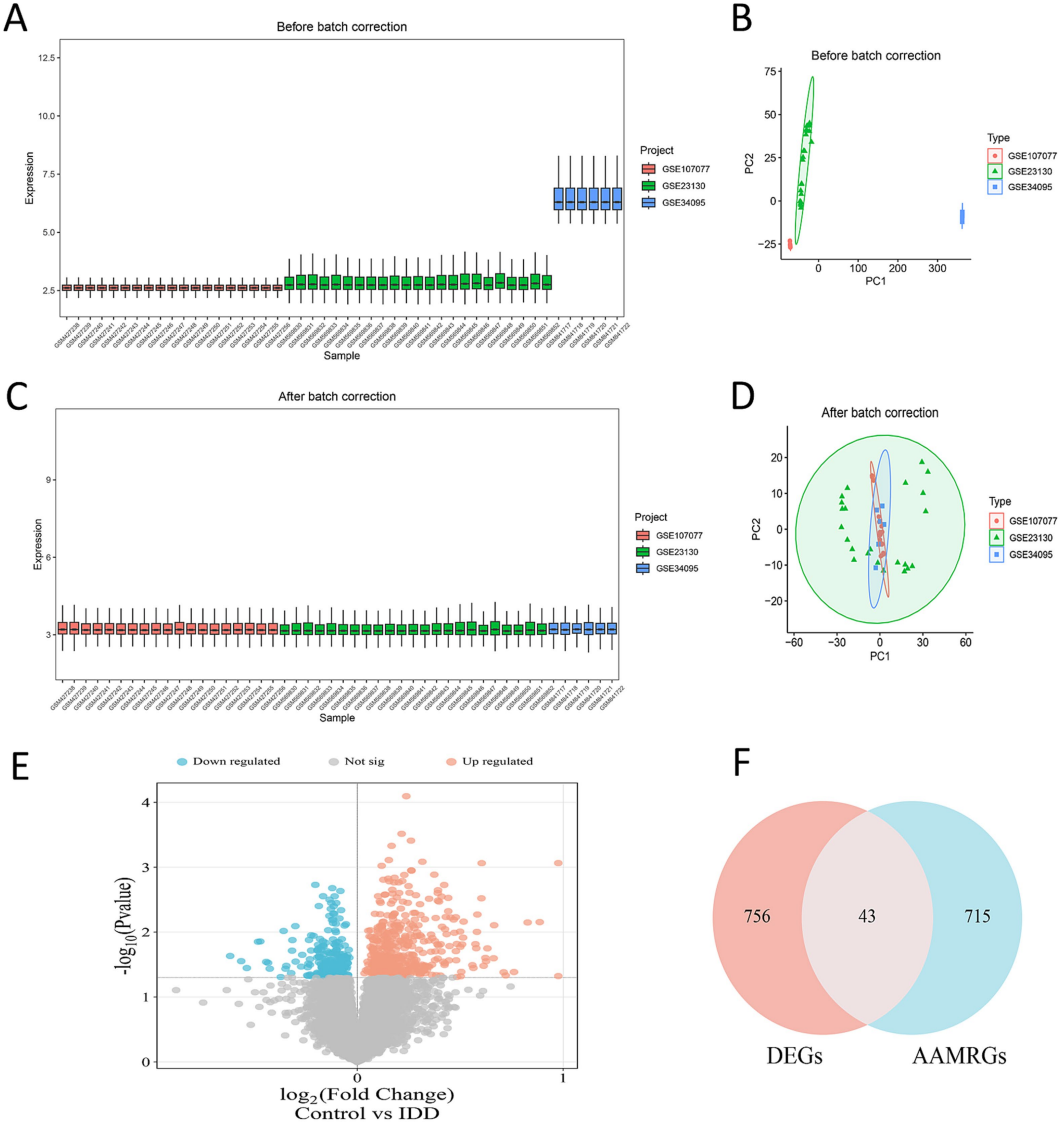
Data integration, screening of differentially expressed genes, and identification of amino acid metabolism-related genes. **(A)** Before correction, samples were clearly separated by dataset origin (batch). **(B)** After correction using the ComBat algorithm, samples from different datasets were well mixed, indicating effective removal of technical batch effects. **(C,D)** Principal component analysis further validated the batch correction effect; post-correction, the separation between control and experimental groups primarily reflects biological differences. **(E)** Volcano plot of differentially expressed genes (DEGs): Shows DEGs between severely degenerated and control tissues. Each point represents a gene. Red indicates significantly upregulated genes, blue indicates significantly downregulated genes, and gray indicates non-significant genes. The dashed lines represent thresholds at log2(FC) = 0 and *p* = 0.05. **(F)** Venn diagram for screening the core gene set: Illustrates the intersection between DEGs and amino acid metabolism-related genes, resulting in the final identification of 43 amino acid metabolism-related differentially expressed genes.

After dataset integration, batch effect correction was performed in the R environment (Figures 2A–D) to eliminate technical bias from different study platforms, ensuring comparability for subsequent analysis. Subsequently, differential expression analysis using the “limma” package with a threshold of *p* < 0.05 identified a total of 663 differentially expressed genes (DEGs) between severely degenerated and control tissues (Supplementary Figure S1, a heatmap showing expression patterns of some significant DEGs; Figure 2E, a volcano plot visualizing the overall distribution of DEGs).

To investigate the potential role of amino acid metabolism in IDD, we obtained 758 amino acid metabolism-related genes (AAMGs) from the GeneCards database (filtering criteria: related to “amino acid metabolism”, protein-coding, relevance score >30). Intersecting these 758 AAMGs with the 663 DEGs yielded 43 amino acid metabolism-related DEGs in intervertebral disk degeneration (Figure 2F). These 43 genes formed the core set for subsequent functional analysis and key gene screening.

### 3.2 Functional enrichment analysis of amino acid metabolism-related DEGs (AAMDEGs)

**Figure 3:**
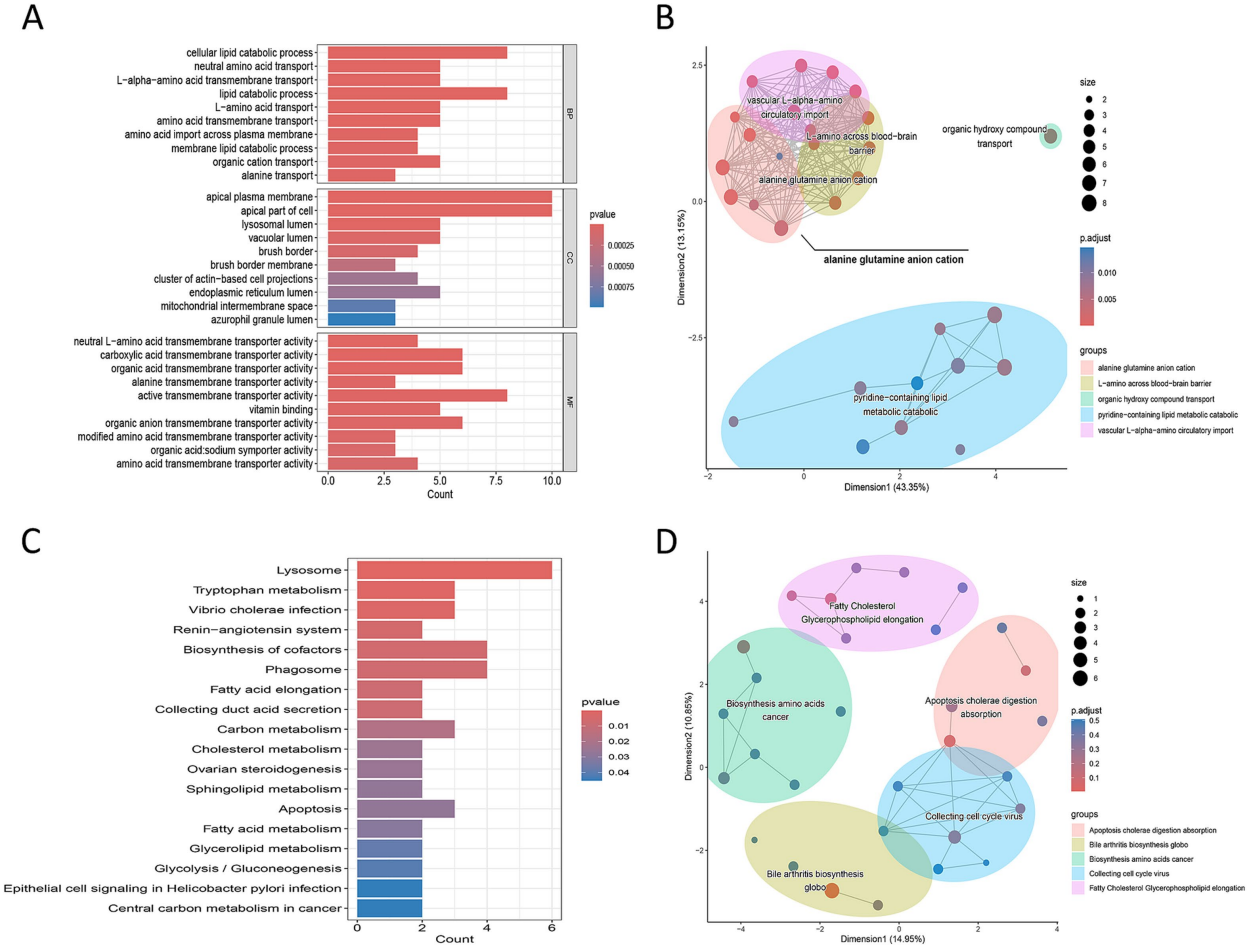
Functional enrichment analysis of amino acid metabolism-related differentially expressed genes. **(A)** Bar graph showing the most significantly enriched Gene Ontology (GO) terms across Biological Process, Cellular Component, and Molecular Function categories. **(B)** Network diagram displaying the top 5 enriched GO processes. **(C)** Bar graph showing significantly enriched Kyoto Encyclopedia of Genes and Genomes (KEGG) pathways. **(D)** Network diagram displaying the top 5 enriched KEGG pathways. The results indicate that the genes are primarily enriched in pathways such as tryptophan metabolism and fatty acid metabolism.

GO enrichment analysis (Figures 3A,B and Supplementary Table S1) showed that the 43 DEGs were mainly enriched in biological processes (BP) such as neutral amino acid transport, L-alpha-amino acid transmembrane transport, L-amino acid transport, and amino acid transmembrane transport; in cellular components (CC), they were significantly enriched in brush border membrane, apical plasma membrane, and lysosomal lumen; in molecular function (MF), they were concentrated in neutral L-amino acid transmembrane transporter activity, alanine transmembrane transporter activity, and organic acid: sodium symporter activity. KEGG pathway enrichment analysis (Figures 3C,D and Supplementary Table S2) indicated that the 43 genes were significantly mapped to biological pathways including tryptophan metabolism, fatty acid metabolism, and amino acid biosynthesis. This suggests that amino acid metabolic imbalance may participate in the pathological process of IDD through multiple pathways.

### 3.3 Identification of amino acid metabolism-related core genes in intervertebral disk degeneration

**Figure 4:**
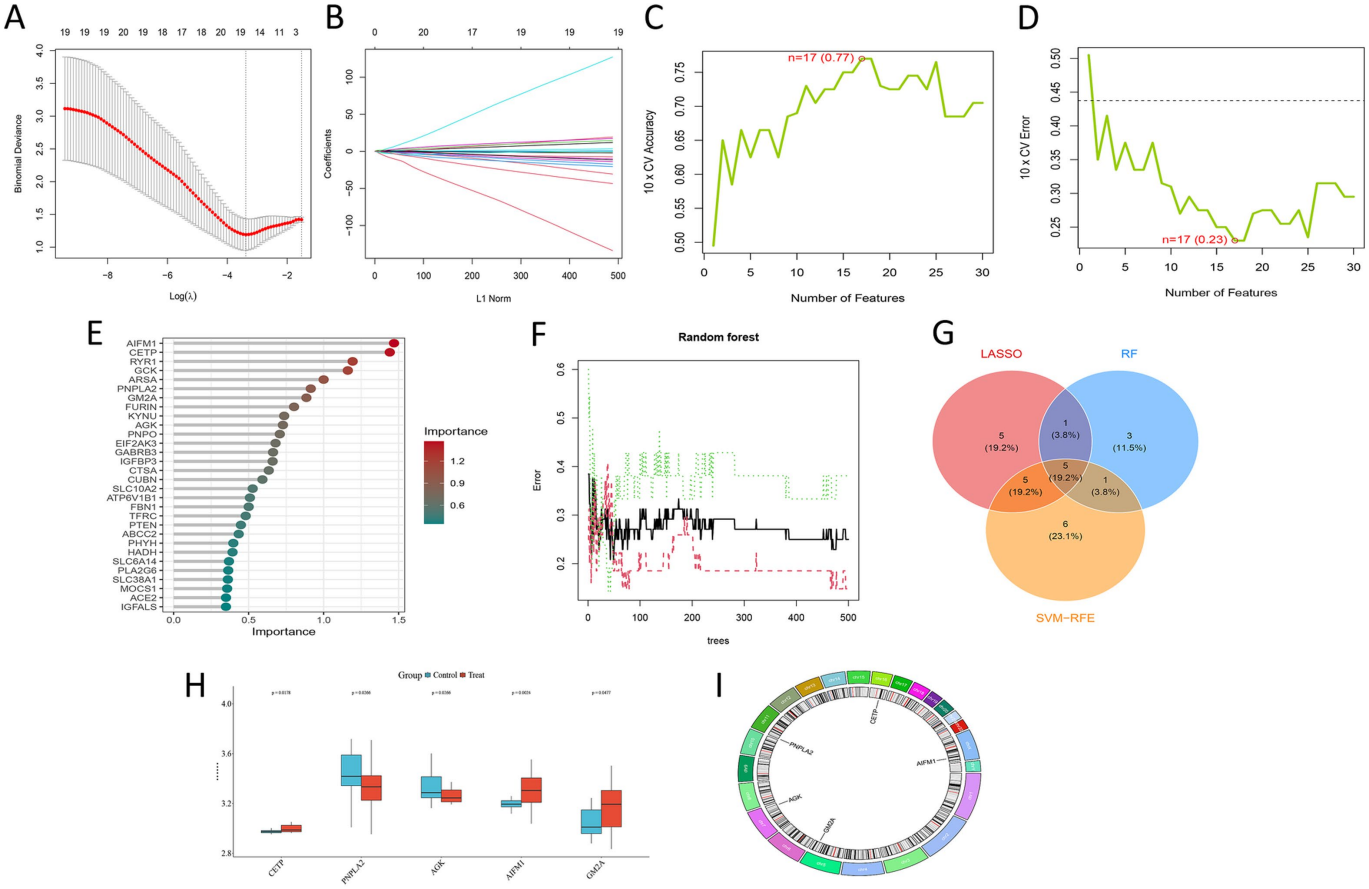
Screening of core genes using multiple machine learning algorithms. **(A)** LASSO coefficient path plot, showing the process of gene coefficients shrinking toward zero as the regularization parameter (Lambda) increases. **(B)** Ten-fold cross-validation error curve. The dashed line indicates the optimal Lambda value corresponding to the minimum error, at which point 16 genes were selected. **(C)** Ten-fold cross-validation accuracy curve across different numbers of features. The peak corresponds to the optimal feature subset. **(D)** The corresponding cross-validation error curve. The minimum error point was used to determine 17 key genes. **(E)** Random Forest- based feature importance ranking plot, illustrating the feature importance scores of all input genes as evaluated by the Random Forest model. **(F)** The model’s error rate decreases and stabilizes as the number of decision trees increases. **(G)** Venn diagram of core gene intersection: Shows the results from LASSO, SVM-RFE, and random forest algorithms. The intersection yields five core genes: CETP, PNPLA2, AGK, AIFM1, and GM2A. **(H)** Box plots of core gene expression: Compares the expression levels of the five core genes between the control and experimental groups. **(I)** Chromosomal localization map of core genes: Shows the specific locations of the five core genes on human chromosomes.

To screen for core amino acid metabolism-related genes associated with disk degeneration, this study employed multiple machine learning methods for feature selection: the LASSO regression model identified 16 candidate genes (Figures 4A,B), the support vector machine model screened 17 genes (Figures 4C,D), and the random forest model selected the top 10 genes based on importance ranking (Figures 4E,F). Taking the intersection of the results from LASSO, SVM-RFE, and random forest yielded five core genes: CETP, PNPLA2, AGK, AIFM1, and GM2A (Figure 4G). Expression analysis results showed that CETP, AIFM1, and GM2A were upregulated in degenerated tissues, while PNPLA2 and AGK were downregulated (Figure 4H). Chromosomal localization (Figure 4I) indicated that CETP is located at 16q21, PNPLA2 at 11p15.5, AGK at 7q34, AIFM1 at Xq26.1, and GM2A at 5q33.1.

### 3.4 Functional enrichment analysis of core genes

To clarify functional and pathway differences between high and low expression groups of the target genes, we performed GSVA enrichment analysis on biological pathways corresponding to the differential expression signatures and visualized the most enriched pathways (Supplementary Figures S2A–E). AIFM1-related pathways: In the high-expression group, pathways such as cell cycle and tRNA biosynthesis were significantly activated; the low-expression group was enriched in energy metabolism-related pathways like glycerophospholipid metabolism and cardiac muscle contraction, suggesting that low AIFM1 expression may regulate IDD by maintaining energy metabolic homeostasis. GM2A-related pathways: The high-expression group was significantly enriched in ECM-receptor interaction and cancer-related signaling pathways (e.g., small cell lung cancer, thyroid cancer), while the low-expression group was enriched in metabolic pathways like purine metabolism and arginine biosynthesis. Combined with the immunomodulatory molecular attribute of GM2A, this suggests it may be involved in IDD progression by regulating energy metabolism and immune-inflammatory responses. CETP-related pathways: The high-expression group was enriched in amino acid metabolism, mismatch repair, etc.; the low-expression group showed activation of glycosylation, dorsal/ventral axis formation, etc., suggesting UCHL1 may influence IDD by regulating cell proliferation, metabolism, and DNA repair. PNPLA2-related pathways: The high-expression group was significantly enriched in histidine metabolism, cardiac muscle contraction, etc.; the low-expression group was enriched in ubiquitin-mediated proteolysis, folate metabolism, etc., suggesting HPGD may promote IDD by regulating lipid metabolism and inflammatory signaling. AGK-related pathways: The high-expression group activated ubiquitin-mediated proteolysis, cell cycle, etc.; the low-expression group was enriched in sugar metabolism, cardiac muscle contraction, etc., further corroborating the synergistic regulatory role of target genes in metabolism, immunity, and matrix remodeling in IDD.

### 3.5 Model interpretation analysis based on SHAP

**Figure 5:**
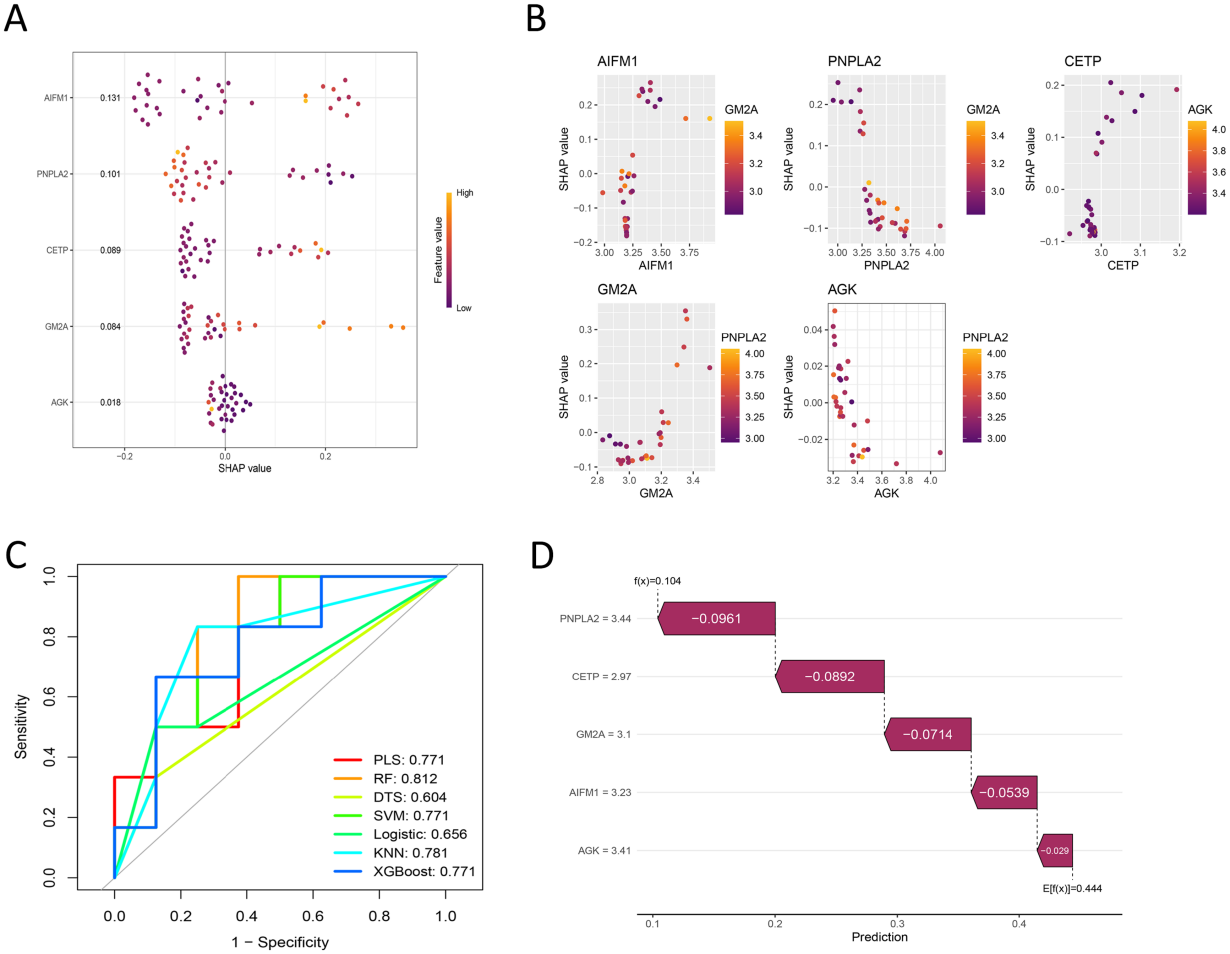
Model interpretability analysis based on SHAP and performance evaluation. **(A)** Bar plot of global gene importance: The *y*-axis represents gene names, and the *x*-axis represents the mean absolute SHAP value. Higher values indicate a greater overall contribution of the gene to the model’s prediction of IDD. **(B)** SHAP dependence plots for individual genes: Show the relationship between the expression level of a single gene and its SHAP value (direction and magnitude of contribution to the model output). Each point represents a sample, and the trend line reveals the nonlinear influence pattern of gene expression. **(C)** ROC curve comparison of machine learning models: Compares the performance of Random Forest, Support Vector Machine, and XGBoost models on the test set. The Random Forest model performed best, with an AUC of 0.812. **(D)** SHAP waterfall plot for individual prediction: Uses a single sample as an example to illustrate how the model cumulatively calculates the final disease risk prediction value from the baseline prediction value (the average output of all samples) based on the SHAP values of the five core genes.

To deeply investigate the role of key genes in model prediction, this study introduced the SHAP method for feature contribution analysis based on machine learning modeling. The SHAP analysis was performed using a random forest model as the explanatory machine learning model, which we selected because it demonstrated the best discriminative ability on the test set (AUC = 0.812). SHAP analysis revealed that genes AGK, GM2A, CETP, PNPLA2, and AIFM1 had key influences on model prediction, with their global SHAP value rankings shown in Figure 5A. Dependency analysis showed a clear nonlinear relationship between the expression levels of these genes and their SHAP values, indicating their expression changes significantly regulate model output (Figure 5B). By constructing models including random forest, support vector machine, and XGBoost, random forest was found to have the best discriminative ability on the test set (Figure 5C). Furthermore, waterfall plots clearly illustrated the cumulative contribution path of each gene feature in individual samples (Figure 5D). In summary, this SHAP analysis systematically quantified the non-linear contributions and interactions of the five core genes in the prediction task, providing an interpretable exploration of gene importance. These findings are complementary to the final logistic regression-based nomogram model, which was constructed for clinical applicability.

### 3.6 Construction and evaluation of the prediction model

**Figure 6:**
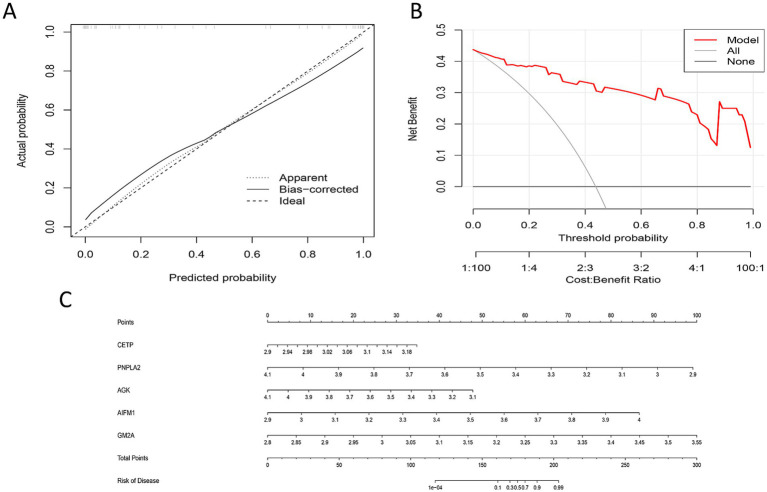
Construction and evaluation of the nomogram diagnostic model based on core genes. **(A)** Model calibration curve: Evaluates the consistency between the model’s predicted risk and the actually observed risk. The black solid line (Apparent) represents the model’s performance on the original data, the gray dashed line (Bias-corrected) represents the performance after Bootstrap bias correction, and the black dashed line (Ideal) represents the perfect diagonal. Curves closer to the diagonal indicate better calibration. **(B)** Decision curve analysis: Evaluates the clinical net benefit of using the model at different threshold probabilities. The red solid line represents the net benefit of using this model to guide intervention strategy, the gray solid line represents the “treat all” strategy, and the black solid line represents the “treat none” strategy. The red curve is higher than the gray and black curves across a wide range of thresholds, indicating good clinical utility of the model. **(C)** Nomogram: Used for individualized risk prediction. Users determine “Points” on the corresponding axis based on the expression levels of the five core genes in a sample, sum all points to obtain the “Total Points,” and then find the corresponding disease probability on the “Risk” axis at the bottom.

Based on the five previously identified key genes (CETP, PNPLA2, AGK, AIFM1, and GM2A), this study used the rms package to construct a nomogram model for predicting the risk of intervertebral disk herniation. This nomogram represents the final clinical prediction tool, designed to utilize the small set of screened key genes to construct a clinically user-friendly, visual risk prediction instrument. The prior complex feature selection step ensured the core status of these five genes, while the final choice of simple logistic regression was made to maximize model interpretability and clinical translational potential. Model performance evaluation showed that in the calibration curve, predicted risks and actually observed risks were distributed close to the diagonal (Figure 6A), indicating good calibration capability without significant systematic bias. Decision curve analysis showed that across a wide range of probability thresholds, the clinical net benefit of using this model for decision-making was superior to the “treat all” or “treat none” strategies, demonstrating good clinical applicability and utility (Figure 6B). The model converts the expression levels of each gene into corresponding scores, calculates individual disease risk probability through weighted total score summation, and enables visual assessment of disease risk (Figure 6C). In conclusion, the nomogram model constructed in this study has good predictive accuracy and clinical translation potential and can be used for individualized risk assessment of intervertebral disk herniation.

### 3.7 Single-cell transcriptomic analysis

**Figure 7:**
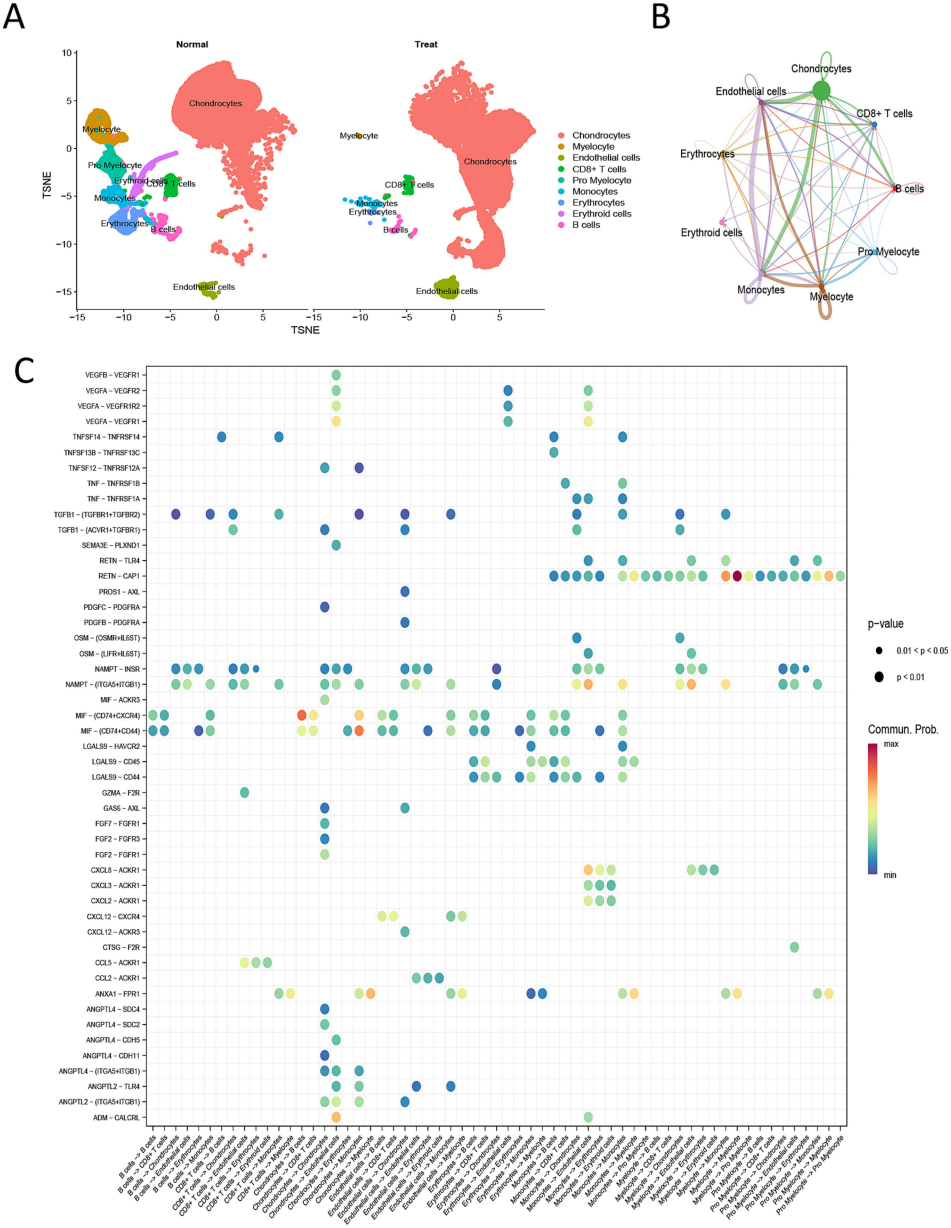
Single-cell transcriptome analysis reveals the IDD microenvironment characteristics. **(A)** t-SNE dimensionality reduction and cell clustering plot: Shows the t-SNE distribution of all cells, with different colors labeling the nine identified cell subpopulations. **(B)** Differential cell–cell communication network plot: Compares the total strength and number of cell–cell communication interactions between normal and degenerated tissues. **(C)** Bubble plot of differential signaling pathway activity: Shows ligand-receptor signaling pathways that are significantly enhanced or weakened in degenerated tissues.

Single-cell transcriptomic analysis constructed a cellular landscape of IDD: After quality control, t-SNE dimensionality reduction visualization clearly presented cellular heterogeneity (Supplementary Figures S3A–D, S4 and Supplementary Table S3); unsupervised clustering identified nine stable cell subpopulations, including chondrocytes, myeloid cells, promyelocytes, endothelial cells, CD8 + T cells, monocytes, erythrocytes, erythroid cells, and B cells (Figure 7A). Analysis of cell subpopulation distribution showed significant differences in the proportion and spatial distribution of cell subpopulations between control and severely degenerated tissues—degenerated tissues had relatively reduced chondrocyte abundance, while infiltration of immune cells like myeloid cells, CD8 + T cells, and monocytes was significantly increased. To analyze changes in cell–cell interactions during degeneration, cell communication analysis was conducted: Compared to normal tissue, the overall strength and complexity of cell–cell communication significantly increased in degenerated nucleus pulposus; myeloid cells, monocytes, and other immune cells served as core signal senders, and communication connections with chondrocytes and endothelial cells were significantly enhanced (Figure 7B, Supplementary Figure S3E and Supplementary Table S4). Analysis of signaling pathways showed that ligand-receptor pairs related to inflammatory responses (e.g., TNF, OSM, CXCL signaling), angiogenesis (e.g., VEGFA signaling), and extracellular matrix remodeling (e.g., TGFB1, FGF signaling) were abnormally active in degenerated tissues (Figure 7C and Supplementary Table S5). This indicates that intervertebral disk degeneration is accompanied by extensive immune cell infiltration and the reconstruction of a pro-inflammatory, pro-catabolic cell communication network, which are key microenvironmental features closely associated with IDD progression.

### 3.8 *In silico* gene knockout analysis

**Figure 8:**
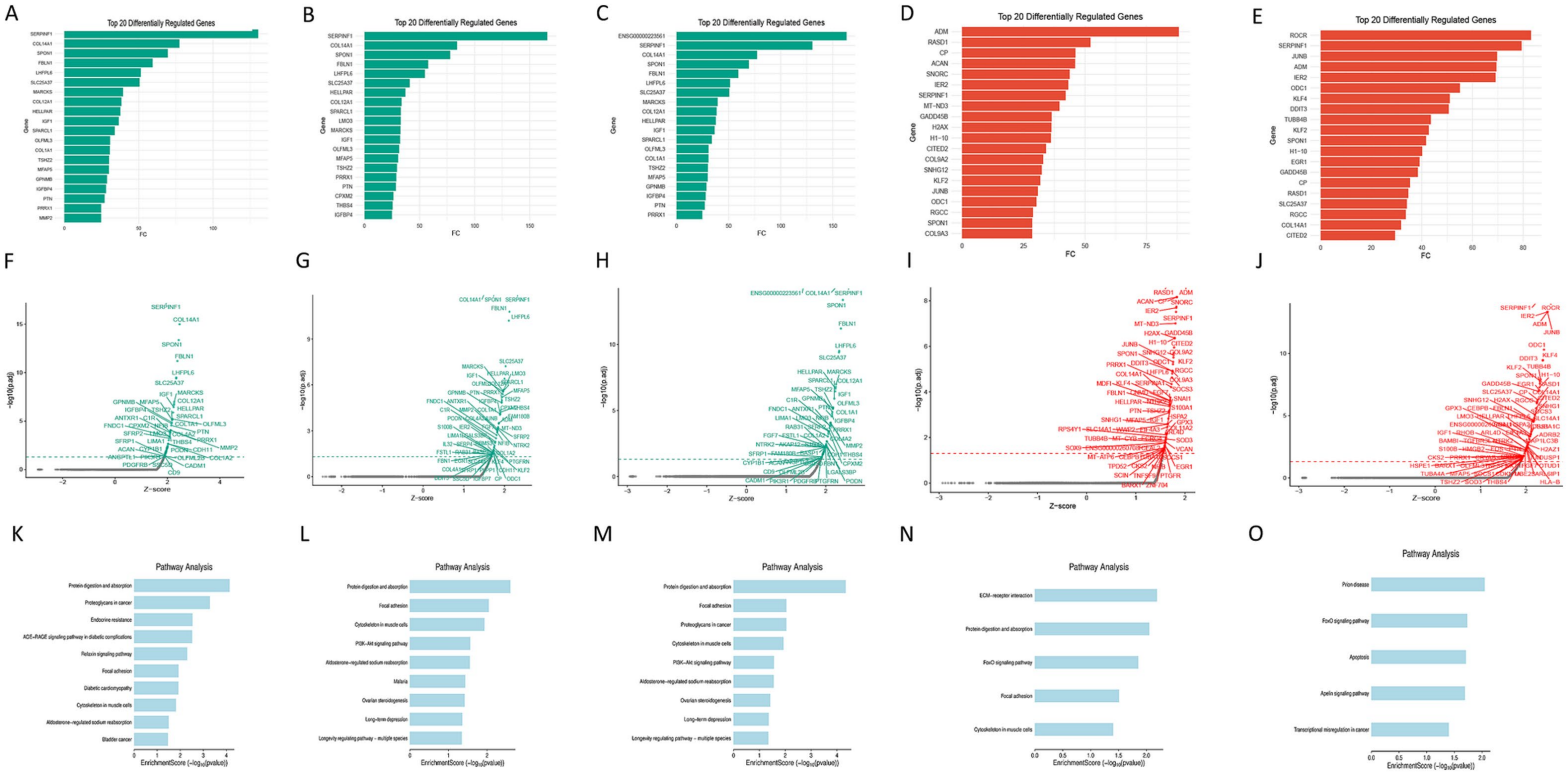
*In silico* knockout simulation of core gene functions. **(A–E)** Heatmaps of key downstream gene expression perturbation: simulate the predicted expression change trends of key downstream effector molecules (SERPINF1, COL14A1, SPON1, IER2) after knocking out each of the five core genes. **(F–J)** Volcano plots of gene expression changes: visually display the magnitude of expression changes for the aforementioned genes after knockout. **(K–O)** Bubble plots of pathway enrichment analysis: show the most significantly enriched biological pathways in the regulatory network after knocking out each core gene.

This study employed the scTenifoldKnk algorithm for in computational prediction method: By simulating the knockout of each of the five core target genes (upregulated genes CETP, AIFM1, GM2A; downregulated genes PNPLA2, AGK) in the single-cell transcriptional network, the impact of their functional loss on the global gene regulatory network was systematically evaluated. Analysis results showed that after knocking out different target genes, the downstream regulatory networks underwent significant specific reprogramming (Supplementary Table S6). After knocking out upregulated genes (CETP, AIFM1, and GM2A): The differentially expressed genes in their regulatory networks were significantly enriched in pathways related to extracellular matrix homeostasis and stress protection. Simultaneously, protective matrix-related genes such as SERPINF1 (pigment epithelium-derived factor), COL14A1 (collagen type XIV), and SPON1 (spondin-1) showed an expression recovery trend, suggesting a relief from suppressed status (Figures 8A–C,F). These computational prediction results suggest that the overexpression of CETP, AIFM1, and GM2A in degeneration may be associated with the inhibition of such protective factors, potentially contributing to ECM degradation and tissue stability disruption. After knocking out downregulated genes (PNPLA2, AGK): Their regulatory networks exhibited stronger characteristics of metabolic disorder and cellular stress. Besides the aforementioned matrix protection genes, the expression of key stress response genes like IER2 (immediate early response 2) was predicted to be further downregulated (Figures 8D,E,I,J). This confirms from the opposite perspective that the functional loss of PNPLA2 and AGK weakens the cell’s ability to cope with metabolic and microenvironmental stress, causing it to lose its “homeostasis maintainer” function and thereby exacerbating the degenerative process. Pathway enrichment results also corroborated this trend. After knocking out upregulated genes, the enrichment levels of ECM repair and stress protection pathways significantly recovered (Figures 8K–M), while after knocking out downregulated genes, the enrichment levels of metabolic disorder and apoptosis-related pathways were further enhanced (Figures 8N,O). These *in silico* predictions provide potential molecular targets and mechanistic hypotheses for subsequent functional validation.

### 3.9 Drug prediction and molecular docking

**Figure 9:**
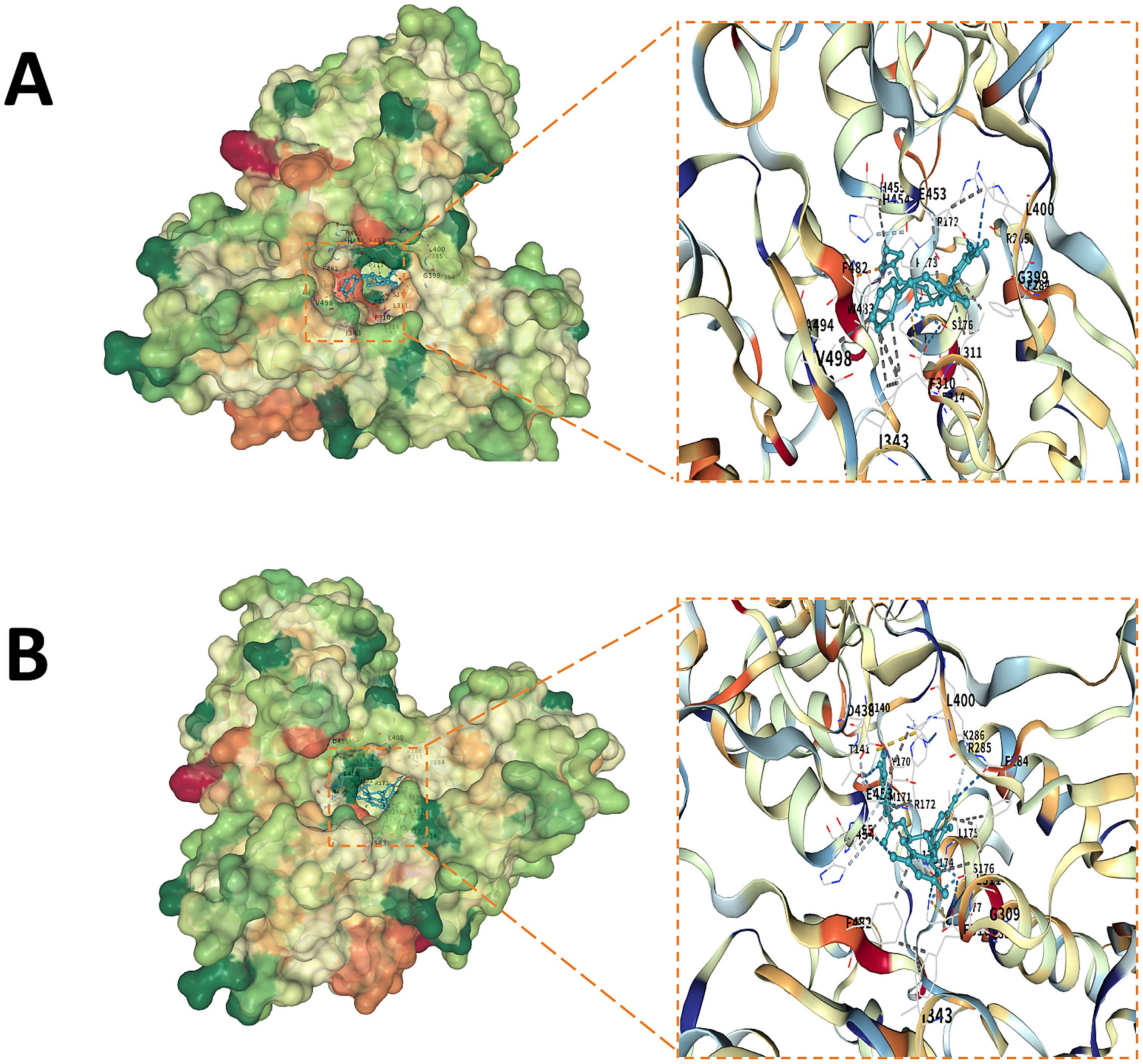
Molecular docking of candidate drugs with core genes. **(A)** Docking mode diagram of NVP-AEW541 with AIFM1: Shows the binding conformation and interactions between the small molecule inhibitor NVP-AEW541 and the target protein AIFM1. **(B)** Docking mode diagram of EGCG with AIFM1: Shows the binding conformation and interactions between the natural compound EGCG and the target protein AIFM1.

Based on drug-gene interaction predictions from DGIdb, we obtained a list of the top 30 candidate drugs related to the five core targets. The complete list of candidate drugs and their corresponding molecular docking binding free energies for all five core genes is provided in Supplementary Table S7. These candidate drugs were subjected to blind docking verification with their corresponding target proteins using the CB-Dock2 platform, and their binding free energy was calculated to assess binding potential. Molecular docking results showed that most candidate drugs exhibited moderate binding affinity to the predicted targets. Among all candidate drugs tested, two compounds demonstrated the strongest binding affinity to the AIFM1 protein: NVP-AEW541, an insulin-like growth factor 1 receptor inhibitor, showed the strongest binding affinity to the target protein AIFM1, with a predicted binding free energy of −10.7 kcal/mol. Epigallocatechin Gallate (EGCG), a polyphenol compound derived from green tea, had a predicted binding free energy of −10.7 kcal/mol to the target protein AIFM1. The docking conformations of these two drugs with their respective targets showed that they could stably bind to potential active pockets or functional regions of the proteins, forming dense hydrogen bonds, hydrophobic interactions, and van der Waals forces (Figures 9A,B). The preliminary docking results indicate that NVP-AEW541 and EGCG exhibit strong binding potential to the AIFM1 protein, suggesting they may serve as lead compounds for targeting AIFM1. However, their effects on AIFM1 function and specific roles in IDD remain to be validated by subsequent cellular and animal experiments.

### 3.10 WB experimental validation

**Figure 10:**
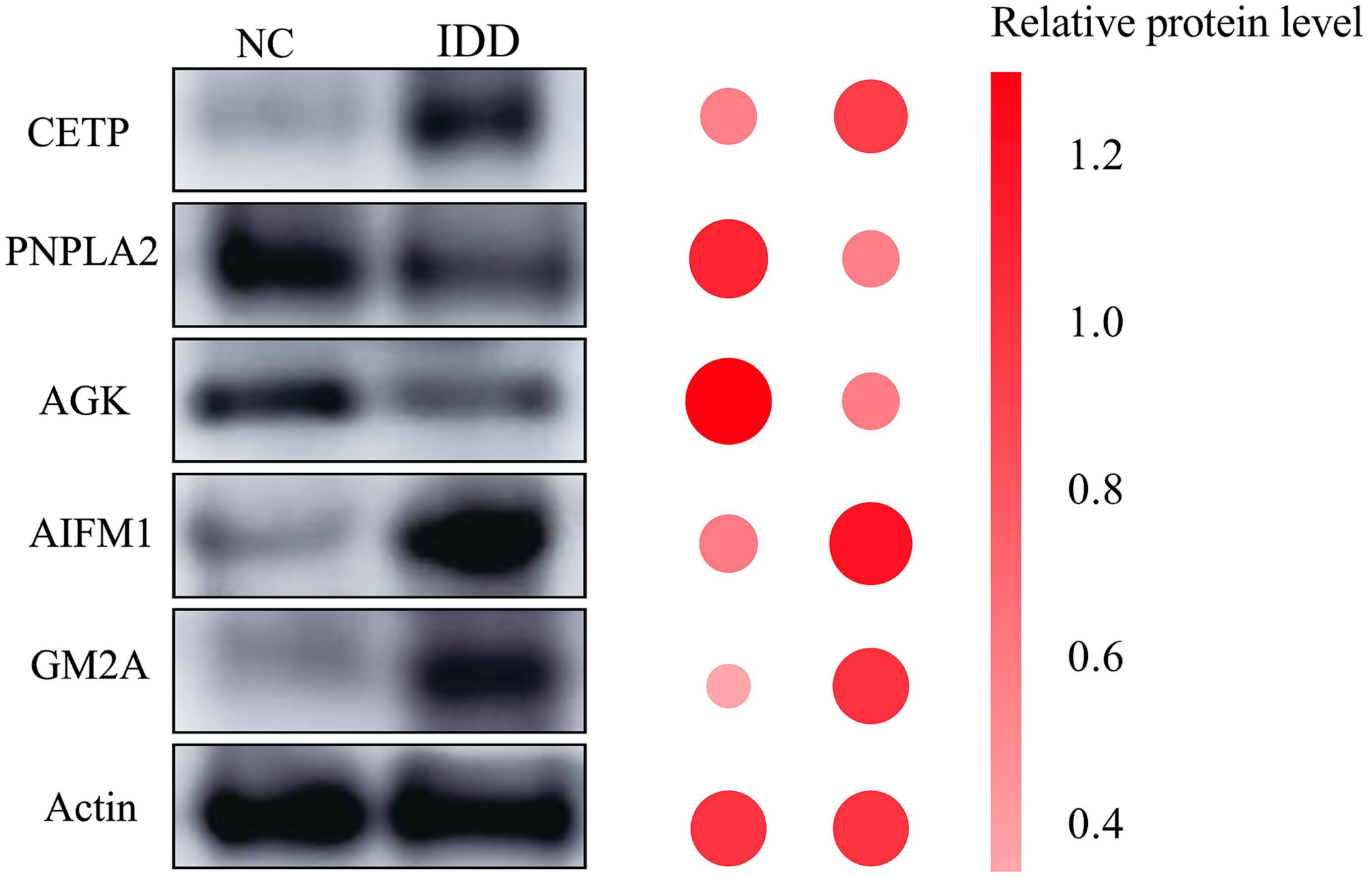
Experimental validation of core gene expression by Western Blot. Western Blot bands: Show the expression of the five target proteins (CETP, PNPLA2, AGK, AIFM1, and GM2A) and the internal reference Actin in the normal control group and the intervertebral disk degeneration group. Quantitative analysis of band gray values is presented, with data shown as mean ± standard deviation. Compared with the normal control group, the protein expression levels of CETP, AIFM1, and GM2A were significantly upregulated in the degeneration group, while the expression levels of PNPLA2 and AGK were significantly downregulated.

To validate the expression patterns of the five core metabolic genes screened by bioinformatics at the protein level, we performed Western Blot analysis on clinical intervertebral disk degeneration tissue samples. As shown in Figure 10, representative Western Blot bands visually displayed the expression differences of the five target proteins between the control and degeneration groups. Quantitative statistical analysis of band grayscale values confirmed that compared to the Pfirrmann grade I-II control group, in the Pfirrmann grade III-V degenerated nucleus pulposus tissues: the protein expression levels of CETP, AIFM1, and GM2A were significantly upregulated, while the protein expression levels of PNPLA2 and AGK were significantly downregulated (all *p* < 0.05).

## 4 Discussion

Intervertebral disk degeneration (IDD) is a leading cause of chronic low back pain, yet the role of cellular metabolic reprogramming in its pathogenesis remains incompletely understood (33, 34). In this study, by integrating multi-omics data, machine learning algorithms, and experimental validation, we identified five core metabolic genes—CETP, AIFM1, GM2A (upregulated) and PNPLA2, AGK (downregulated)—that are strongly associated with IDD. These genes may constitute a coordinated regulatory network implicated in metabolic imbalance, cellular stress, and microenvironment remodeling.

The five core genes suggest a state of metabolic dysregulation in degenerated nucleus pulposus cells. PNPLA2, responsible for lipid breakdown, and AGK, involved in mitochondrial membrane lipid synthesis, were both downregulated. Similar observations have been reported in osteoarthritis, reflecting that under degenerative stress, cells may become unable to effectively mobilize stored lipids for energy or maintain mitochondrial integrity (35–37). Concurrently, the upregulation of CETP and GM2A suggests dysregulation of cell membrane lipid metabolism, which has been associated with inflammasome activation and altered signal transduction (38, 39). These findings collectively depict a complex landscape of metabolic remodeling in IDD, revealing abnormal expression patterns of core metabolic genes and their strong correlation with disease progression.

AIFM1, a key executor of mitochondrial apoptosis, was significantly upregulated in degenerated tissues. This positions AIFM1 as a potential link between upstream metabolic stress and downstream cell fate (40, 41). Our computational *in silico* knockout predictions suggested that the upregulation of CETP, AIFM1, and GM2A may suppress protective matrix genes such as SERPINF1, SPON1, and COL14A1. SERPINF1 is known to possess anti-inflammatory and matrix-protective properties (42), while COL14A1 contributes to ECM mechanical stability (43). Conversely, the predicted functional loss of PNPLA2 and AGK may weaken cellular energy supply and stress response capacity, potentially impairing the activation of stress-responsive genes like IER2 (44, 45). These computational predictions provide testable hypotheses regarding a potential pathological cascade from metabolic dysregulation to protective barrier failure (46).

Our single-cell transcriptomic analysis revealed that IDD is accompanied by significant immune cell infiltration (e.g., myeloid cells, monocytes, CD8 + T cells) and an enhanced cell–cell communication network. This aligns with current research on the immune-inflammatory microenvironment in IDD (47, 48). Notably, signaling pathways involving TNF, VEGFA, and TGFB1 were abnormally active in degenerated tissues. We hypothesize that metabolic gene dysregulation may alter the secretory profile of nucleus pulposus cells, thereby recruiting and activating immune cells (49, 50). Activated immune cells may further accelerate ECM degradation via matrix-degrading enzymes and pro-inflammatory factors, potentially establishing a positive feedback loop (51, 52). Activated immune cells (e.g., monocytes/macrophages) further accelerate ECM destruction by releasing matrix-degrading enzymes like MMP9 and pro-inflammatory factors, forming a “metabolic abnormality-cell damage-immune infiltration-inflammation amplification” positive feedback vicious cycle (53). This may help explain why a chronic low-grade inflammatory state persists within degenerated disks.

From a translational perspective, the nomogram diagnostic model based on the five core genes showed good predictive performance (AUC: 0.812). This provides a candidate biomarker combination for early IDD diagnosis. Additionally, through drug-gene interaction screening and molecular docking, we found that NVP-AEW541 and epigallocatechin gallate (EGCG) exhibited high binding affinity to AIFM1 (both −10.7 kcal/mol) (54–56). EGCG has been reported to have protective effects in spinal cord injury and osteoarthritis (57). However, it is important to note that these findings are based on computational predictions; their efficacy and safety require systematic validation through future *in vitro* and *in vivo* pharmacological studies. Our findings suggest AIFM1 as a potential target warranting further investigation. These observations collectively suggest that targeting the upregulated genes or enhancing the function of downregulated genes may represent potential strategies for IDD intervention. Overall, our findings reveal abnormal expression patterns of core metabolic genes in IDD and their strong correlation with disease progression, providing a basis for hypothesis generation that requires functional experiments to validate causal roles.

This study has several limitations. First, the causal relationships suggested by our findings require validation through in vitro functional experiments (e.g., gene knockdown/overexpression) and animal models. The computational predictions, including *in silico* knockout and drug docking, are hypothesis-generating rather than mechanism-validating. Second, the study is primarily based on transcriptomic and proteomic data; metabolomic analyses would be valuable to directly assess metabolite-level changes. Third, the predicted drug targets await confirmation via cellular and pharmacological studies, and none of the findings have been validated in animal models. Future studies will focus on the most promising targets, such as AIFM1 and PNPLA2, using in vitro and in vivo models to conduct in-depth functional validation, aiming to confirm their specific roles in the initiation and progression of IDD. Future studies using appropriate IDD animal models are needed to evaluate the in vivo efficacy and safety of targeting these genes or using the predicted compounds.

## 5 Conclusion

In summary, by integrating multi-omics data analysis, multiple machine learning algorithms, and experimental validation, this study identified five core metabolic genes (CETP, AIFM1, GM2A, PNPLA2, and AGK) that are strongly associated with intervertebral disk degeneration. These genes may constitute a coordinated regulatory network linking metabolic dysregulation, cellular stress, and microenvironment remodeling, providing a mechanistic hypothesis for the pathogenesis of IDD. The constructed nomogram prediction model (AUC: 0.812) shows promising diagnostic potential, and drug virtual screening (e.g., NVP-AEW541 and EGCG) provides preliminary leads for targeted therapy. In conclusion, this study offers new insights into the potential metabolic mechanisms underlying IDD and provides a theoretical foundation for developing early diagnostic tools and targeted intervention strategies that warrant further validation.

## Data Availability

The datasets presented in this study can be found in online repositories. The names of the repository/repositories and accession number(s) can be found in the article/Supplementary material.

## Footnotes

1
https://www.genecards.org/

2
http://183.56.231.194:8001/cb-dock2/index.php
