# Supplementary material for: Integrating multi-omics and machine learning to explore the role of amino acid metabolism in intervertebral disk degeneration
Source: Front Neurol. 2026 May 13;17:1808282. doi: 10.3389/fneur.2026.1808282 (PMC13212091; doi:10.3389/fneur.2026.1808282)
Supplement: Supplementary file 8 [file Supplementary_file_1.docx]

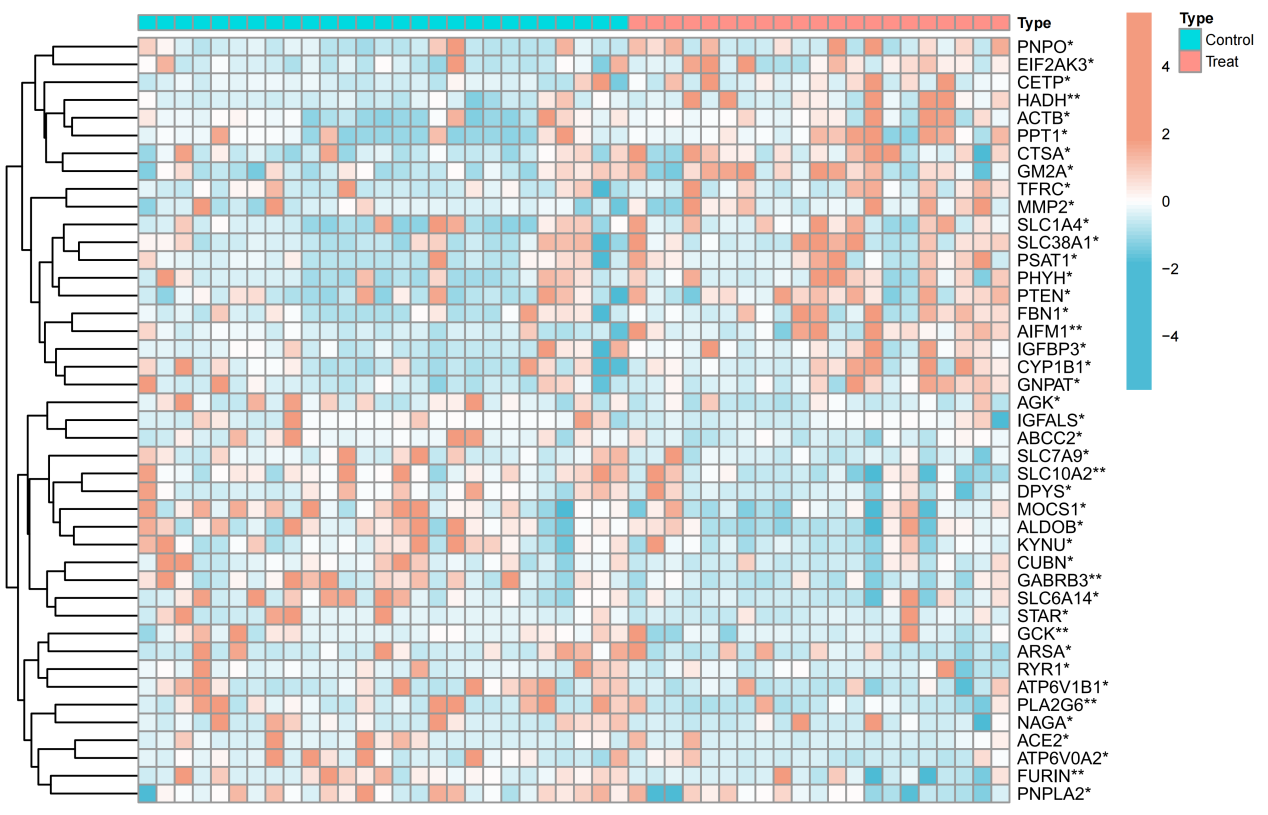


**Figure S1.** Heatmap of Differentially Expressed Genes.The heatmap visualizes the expression patterns of a subset of the most significantly differentially expressed genes across all samples. Each row represents an individual gene, and each column represents a sample. The color scale indicates the normalized expression levels, with red signifying high expression and blue signifying low expression.


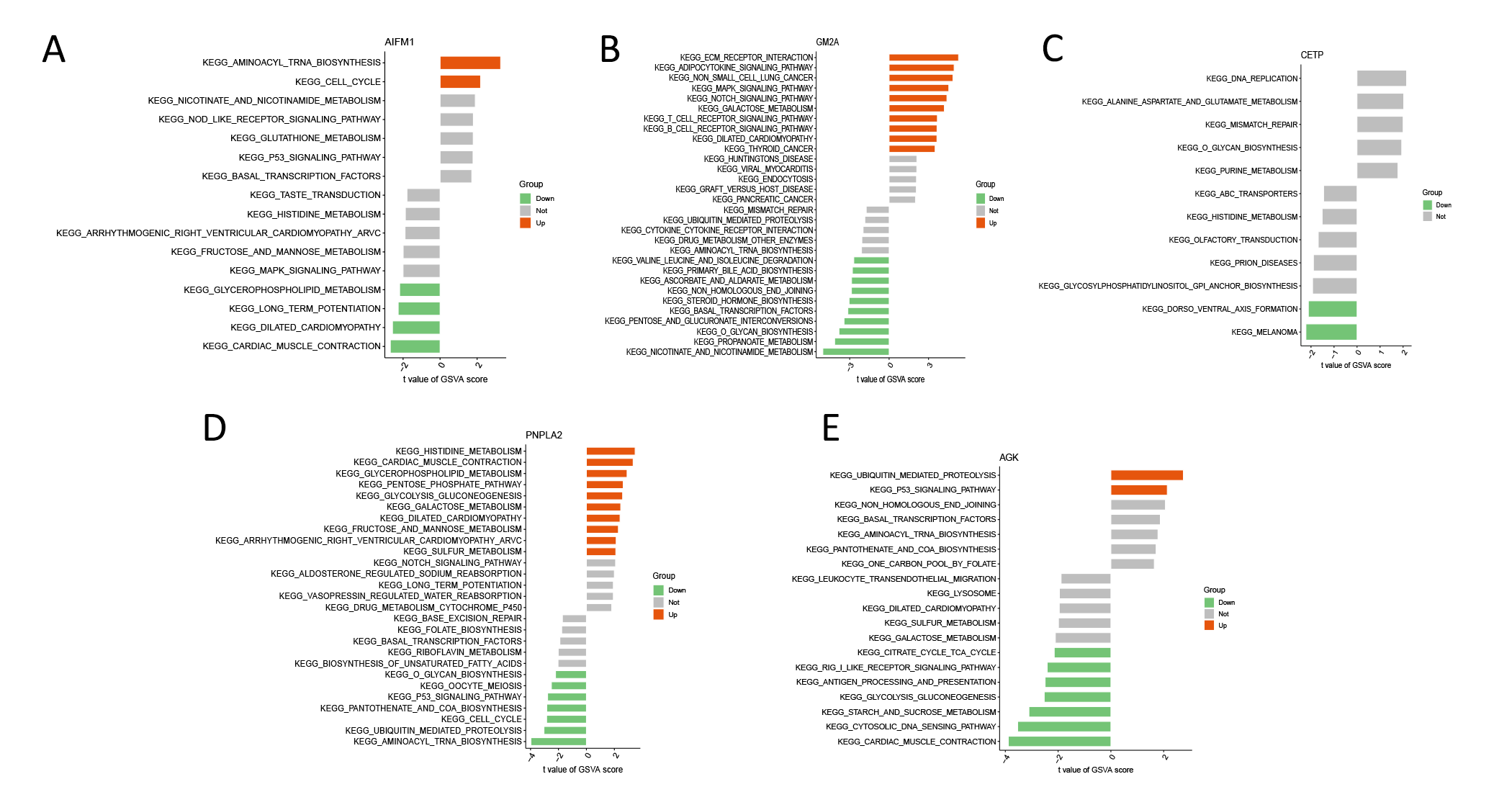


**Figure S2.** GSVA-Based Functional Enrichment Analysis of Core Genes. A-E. Heatmaps of Pathway Enrichment in High- and Low-Expression Groups for Each Core Gene: These panels display the Gene Set Variation Analysis (GSVA) enrichment scores for representative biological pathways. Samples are grouped based on their expression levels of the respective core genes: CETP (A), PNPLA2 (B), AGK (C), AIFM1 (D), and GM2A (E). In the heatmaps, red indicates pathway activation, while green indicates pathway suppression.。


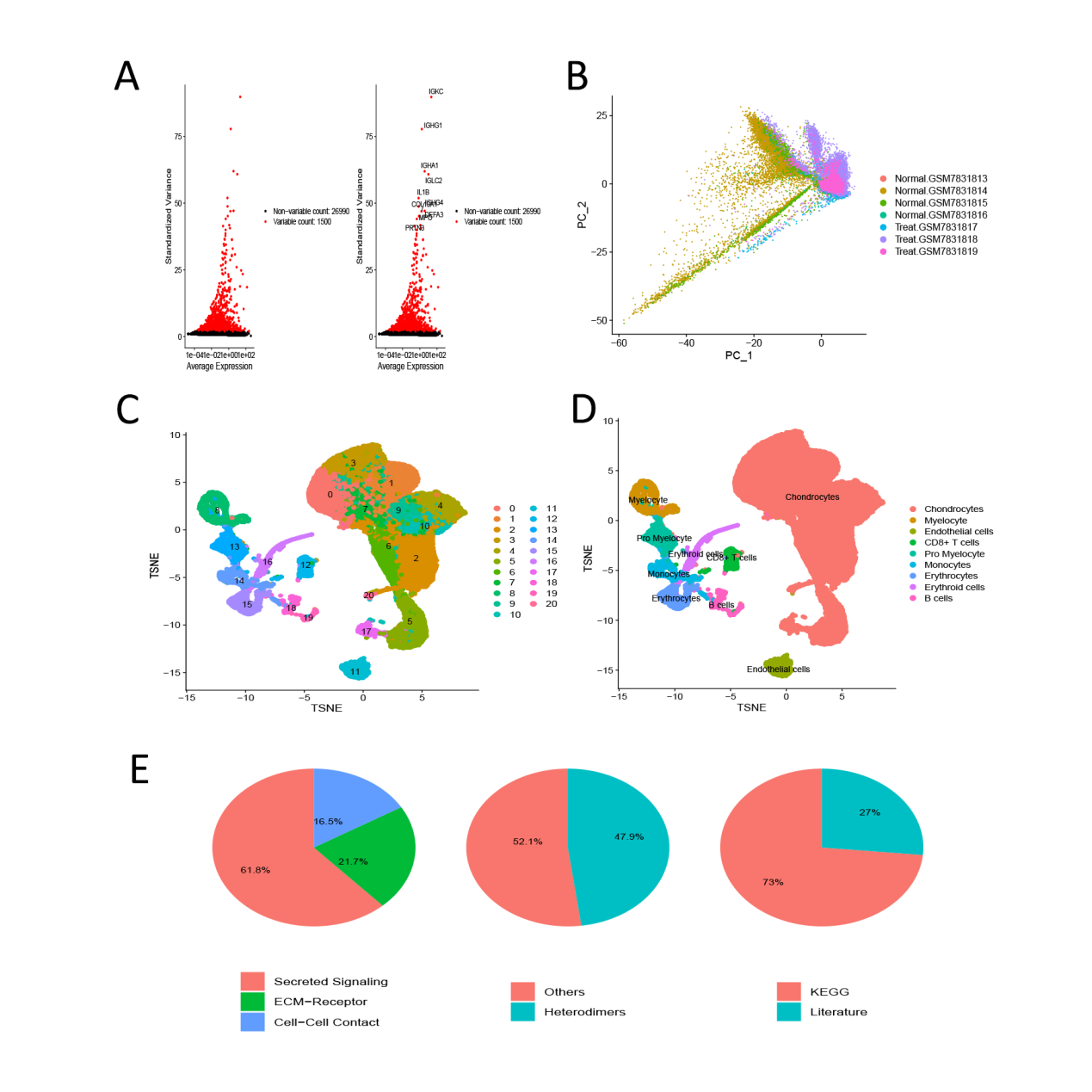


**Figure S3.** Quality Control and Preliminary Analysis of Single-Cell RNA Sequencing Data. A-D. Data Quality Control Plots: These panels present key quality control metrics, including the distribution of detected genes per cell and the percentage of mitochondrial gene content per cell. E. Sources of Cell-Cell Communication Evidence.


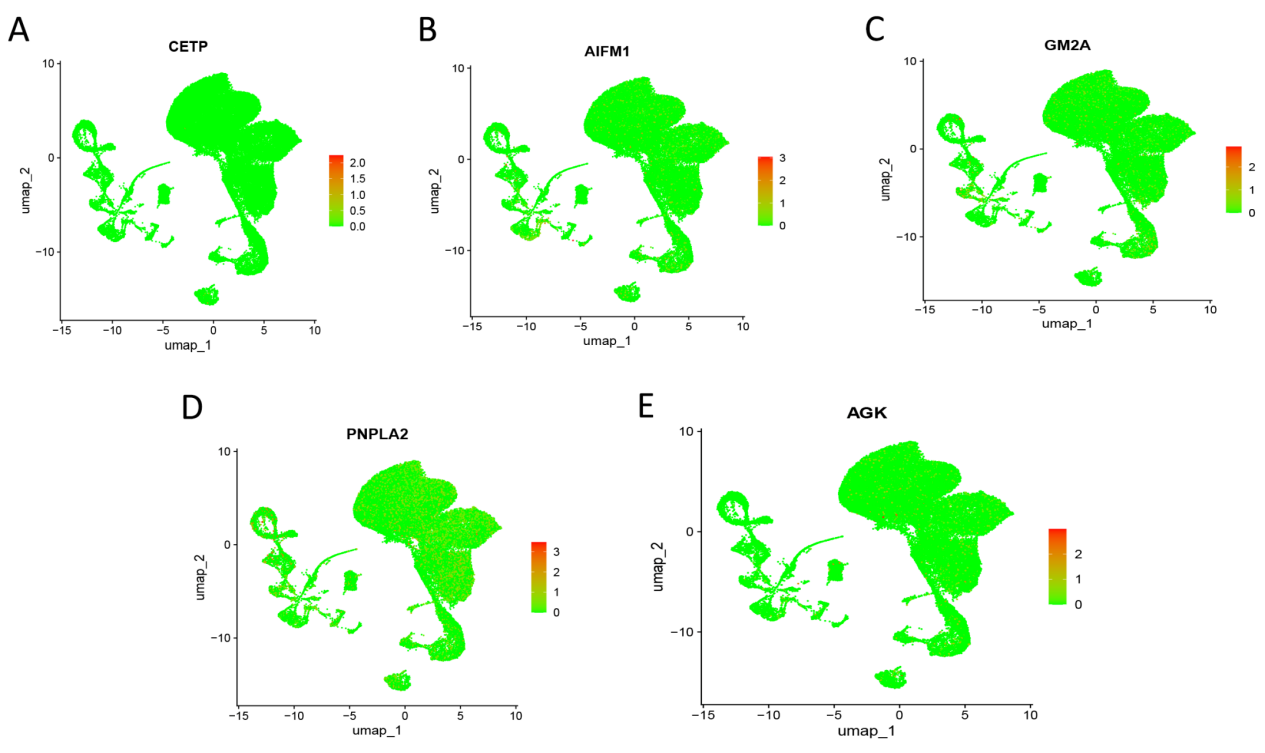


**Figure S4.** UMAP Visualization of the Expression Distribution of Five Core Metabolic Genes across Disc Cell Subpopulations. A-E. Expression Feature Plots: Panels (A-E) display the UMAP projections illustrating the expression distribution of the five core metabolic genes (CETP, AIFM1, GM2A, PNPLA2, and AGK) across the identified intervertebral disc cell subpopulations.
